# Unveiling the Mechanisms for Decreased Glutathione in Individuals with HIV Infection

**DOI:** 10.1155/2012/734125

**Published:** 2011-12-29

**Authors:** Devin Morris, Carlos Guerra, Clare Donohue, Hyoung Oh, Melissa Khurasany, Vishwanath Venketaraman

**Affiliations:** ^1^Graduate College of Biomedical Sciences, Western University of Health Sciences, Pomona, CA 91766, USA; ^2^Science Department, College of Osteopathic Medicine of the Pacific, Western University of Health Sciences, Pomona, CA 91766, USA; ^3^Pitzer College, 1050 N Mills Avenue, Claremont, CA 91711, USA; ^4^College of Dental Medicine of the Pacific, Western University of Health Sciences, Pomona, CA 91766, USA; ^5^Department of Basic Medical Sciences, College of Osteopathic Medicine of the Pacific, Western University of Health Sciences, 309 East Second Street, Pomona, CA 91766, USA

## Abstract

We examined the causes for decreased glutathione (GSH) in individuals with HIV infection. We observed lower levels of intracellular GSH in macrophages from individuals with HIV compared to healthy subjects. Further, the GSH composition found in macrophages from HIV^+^ subjects heavily favors oxidized glutathione (GSSG) which lacks antioxidant activity, over free GSH which is responsible for GSH's antioxidant activity. This decrease correlated with an increase in the growth of *Mycobacterium tuberculosis (M. tb)* in macrophages from HIV^+^ individuals. In addition, we observed increased levels of free radicals, interleukin-1 (IL-1), interleukin-17 (IL-17) and transforming growth factor-*β* (TGF-*β*) in plasma samples derived from HIV^+^ individuals compared to healthy subjects. We observed decreased expression of the genes coding for enzymes responsible for de novo synthesis of GSH in macrophages derived from HIV^+^ subjects using quantitative PCR (qPCR). Our results indicate that overproduction of proinflammatory cytokines in HIV^+^ individuals lead to increased production of free radicals. This combined with the decreased expression of GSH synthesis enzymes leads to a depletion of free GSH and may lead in part to the loss of immune function observed in HIV patients.

## 1. Introduction

According to the World Health Organization (WHO), it is estimated that approximately 33.3 million people are infected with HIV, two-thirds of which live in sub-Saharan Africa. It is also estimated that nearly 2.6 people are infected each year [1]. People diagnosed with AIDS often suffer from life-threatening diseases caused by opportunistic infections including tuberculosis (TB). In recent years, there has been a significant increase in the incidence of TB due to the emergence of multidrug and extreme-drug resistant strains of *M. tb* and due to increased numbers of highly susceptible immunocompromised individuals arising from the AIDS pandemic. It is also believed that in developing countries, as many as 40 to 80% of individuals with AIDS are at risk of developing TB [2, 3]. In previous studies, our lab has reported that the virulent strain of *M. tb* is sensitive to the antioxidant, GSH [4–9]. Our studies have shown that HIV-infected individuals have deficiencies of intracellular GSH in red blood cells (RBC's) as well as peripheral blood mononuclear cells (PBMC's), which include T cells, natural killer cells, and monocytes [8]. 

GSH is a tripeptide composed of glutamine, cysteine, and glycine which plays a major role in the maintenance of the intracellular redox state. GSH is also important for cellular homeostasis as well as many different cellular functions such as protein synthesis, enzyme catalysis, transmembrane transport, receptor action, intermediary metabolism, and cell maturation [10–12]. GSH is produced by nearly all cell types and exists in two forms. Reduced or free GSH is responsible for the antioxidant functions of GSH, while GSSG is the byproduct of the free radical scavenging activity of GSH and lacks antioxidant function [10–12].

**Figure 1:**
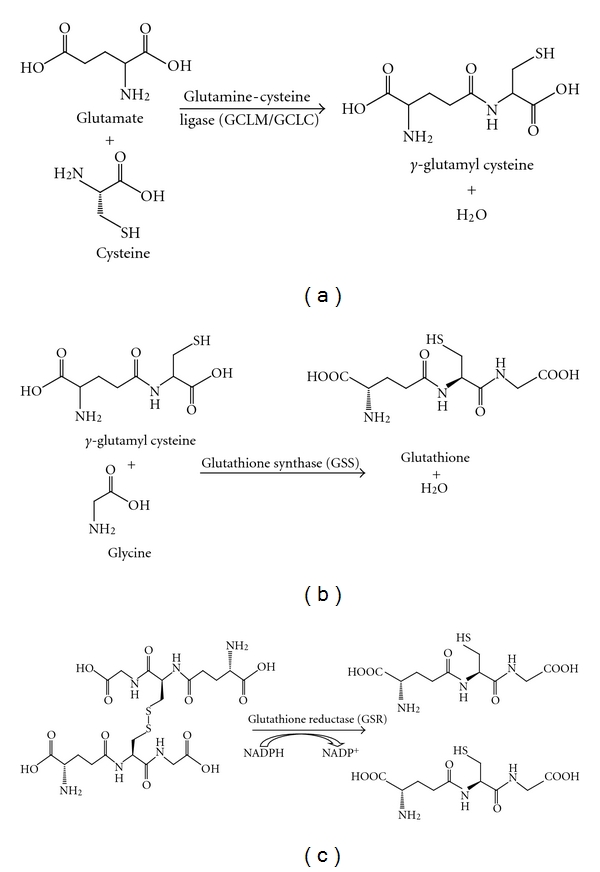
(a) The first step in de novo GSH biosynthesis is rate limiting. Glutamine and cysteine are linked by the homodimeric enzyme glutamine-cysteine ligase, (b) GSS catalyzes the second step in GSH biosynthesis, linking glycine and *γ*-glutamyl cysteine to form GSH. (c) Oxidized GSH can be converted to free GSH by GSR, utilizing NADPH as a cofactor.

Free GSH is synthesized in the cell via two different mechanisms. Free GSH can be synthesized de novo through a two-step process which is mediated by two different enzymes. The first, and rate-limiting step in de novo GSH synthesis is the linking of glutamine and cysteine by glutamine-cysteine ligase (GCL) to form *γ*-glutamyl cysteine (Figure 1(a)). The second step is the linking of glycine to **γ**-glutamyl cysteine which is catalyzed by the enzyme glutathione synthase (GSS) (Figure 1(b)). Free GSH can also be synthesized via the reduction of GSSG. This reaction is catalyzed by glutathione Reductase (GSR) which utilizes NADPH as a cofactor (Figure 1(c)).

It is our hypothesis that chronic HIV infection leads to excessive production of proinflammatory cytokines such as IL-1, IL-17, and TNF-*α*. The chronic overproduction of proinflammatory cytokines leads to the generation of free radicals. These free radicals are scavenged by free GSH. The excessive production of free radicals in HIV-infected individuals will lead to the depletion of GSH. In addition, elevated TGF-*β* blocks the production of GCLC which reduces the production of new molecules of GSH. Elevated levels of IL-1 will also facilitate the loss of intracellular cysteine, which further reduces the production of new GSH molecules. We tested our hypothesis by performing in vitro studies using human monocyte-derived macrophages isolated from healthy individuals and individuals with HIV infection. Our results signify an important mechanism that is responsible for decreasing the levels of GSH in macrophages from individuals with HIV infection resulting in enhanced multiplication and survival of intracellular *M. tb*.

## 2. Materials and Methods

### 2.1. Subjects

A total of 26 volunteers (13 healthy subjects and 13 individuals with HIV infection) were recruited for the study. Individuals with HIV infection were recruited from the Foothills AIDS project. Healthy subjects without HIV infection or a history of TB were recruited from the university faculty and staff. All HIV-infected volunteers had been diagnosed with HIV-1, were taking some form of antiretroviral treatment (ART), and had CD4^+^ T-cell counts between 271 and 1415 cells per mm^3^. Thirty five milliliters of blood was drawn once from both healthy volunteers and individuals with HIV infection after obtaining a signed informed consent. All our studies were approved by both the Institutional Review Board and the Institutional biosafety committee.

### 2.2. Isolation of Monocytes and In Vitro Culture for Differentiation to Macrophages

We first isolated PBMC from the whole blood of healthy and HIV-infected individuals using density gradient centrifugation with FICOLL Histopaque (Sigma). Plasma samples from healthy and HIV-infected subjects were collected for cytokine measurement. Monocytes were isolated from PBMCs by adherence to poly-L lysine (%0.005) treated-96 well-tissue culture plates. Briefly, PBMCs (1 × 10^5^ cells/well) were added to poly-L lysine-treated tissue culture plates and incubated overnight at 37°C to facilitate monocyte adherence. Following overnight adherence, the nonadherent cells were removed, and fresh media [RPMI (Sigma) supplemented with 5% human AB serum (Sigma)] was added to the adherent cells which were then incubated for 7 days in culture conditions to allow the monocytes to differentiate into macrophages.

### 2.3. Assay of IL-1, TGF-*β*, IL-17, and Malondialdehyde (MDA) in Plasma and Macrophage Lysates

Cytokines were measured from collected plasma and macrophage culture supernatants by enzyme-linked immunosorbent assay (ELISA) (eBioscience). MDA was measured in macrophage lysates by colorimetric assay (Cayman Chemical).

### 2.4. Assay of GSH Levels in Macrophages from Healthy and HIV-Infected Subjects

GSH levels were measured in isolated macrophages from healthy subjects and individuals with HIV infection. Intracellular levels of GSH in macrophages were determined by spectrophotometry using an assay kit from Arbor Assays. Briefly, macrophages (3 × 10^5^) were detached from the culture plate by treatment with trypsin (50 *μ*L/10^5^ cells) for 10 minutes in culture conditions. Detached cells were washed and resuspended in ice cold 5% 5-sulfosalicylic acid dehydrate solution (SSA). Supernatants collected after centrifugation were analyzed for total and oxidized GSH as per manufacturer's instructions. Free GSH was calculated by subtracting measured oxidized GSH concentrations from the measured total GSH concentrations, per the manufacturer's instructions. All GSH measurements were normalized with total protein levels.

### 2.5. Assay of Total Protein Levels in Macrophage Supernatants and Cell Lysates

Proteins in the isolated macrophage supernatants and lysates were measured by Bradford's method using a Coomassie protein assay reagent (Thermo Scientific).

### 2.6. Preparation of Bacterial Cells for Macrophage Infection

All infection studies were performed using the virulent laboratory strain of *M. tb*, H37Rv inside the biosafety level 3 (BSL-3) facility. *M. tb* was processed for infection as follows: static cultures of H37Rv at their peak logarithmic phase of growth (between 0.5 and 0.8 at A600) were used for infection of monocytes. The bacterial suspension was washed and re-suspended in RPMI (Sigma) containing AB serum (Sigma). Bacterial clumps were disaggregated by vortexing five times with 3 mm sterile glass beads. The bacterial suspension was passed through a 5 *μ*m syringe filter (Millipore) to remove any further clumps. The total number of organisms in the suspension was ascertained by plating. Processed H37Rv was frozen as stocks at −80°C. At the time of infection, frozen stocks of processed H37Rv were thawed and used for monocyte infection.

### 2.7. Infection of Macrophages with *M. tb*

Monocyte-derived macrophages were infected with processed H37Rv at a multiplicity of infection of 10 : 1 and incubated for 2 hours for phagocytosis. Unphagocytosed mycobacteria were removed by washing the infected macrophages three times with warm sterile PBS. Infected macrophages were cultured in RPMI + 5% AB serum. Infected cultures were terminated at 1 hour and 5 days postinfection to determine the intracellular survival of H37Rv. Culture supernatants were collected, filtered through a 0.2 *μ*m filter, and stored at −80°C for the assay of cytokines.

### 2.8. Termination of Infected Macrophage Cultures and Measurement of Colony Forming Units

The termination of *M. tb *infected cultures was performed by the addition of 200 *μ*L of distilled sterile water to each culture well. The collected cell lysates were plated on 7H11 medium (Hi Media) enriched with albumin dextrose complex (ADC), to estimate the extent of H37Rv growth in macrophages.

### 2.9. Quantitative PCR Analysis of GSH Synthesis Genes

RNA was isolated from macrophages from healthy and HIV-infected individuals using Trizol (Invitrogen) per the manufacturer's instructions. Isolated RNA was quantified using a NanoVue spectrophotometer (GE). Isolated RNA was reverse transcribed to cDNA using a qScript cDNA synthesis kit (Quanta Biosciences) per the manufacturer's instructions. qPCR analysis of gene expression was performed using an EvaGreen qPCR master mix (Biotium) and primers for the GSH-metabolism genes: glutamine-cysteine ligase (catalytic subunit) (GCLC), glutamine-cysteine ligase (modulatory subunit) (GCLM), glutathione synthetase (GSS), glutathione Reductase (GSR), and *γ*-glutamyl transferase 1 (GGT1) (Elim Biopharmaceuticals) on a StepOne Plus thermocycler (ABI) per the manufacturer's instructions. Using the ΔΔCt method, comparative gene expression analysis was performed. Target gene expression was compared to the endogenous control gene *β*-actin (ACTB) for each sample. Gene expression for each target gene in samples from HIV-infected subjects was compared to the expression observed in healthy controls.

## 3. Results

### 3.1. Assay of Cytokines and MDA in Plasma and Macrophage Supernatants

**Figure 2:**
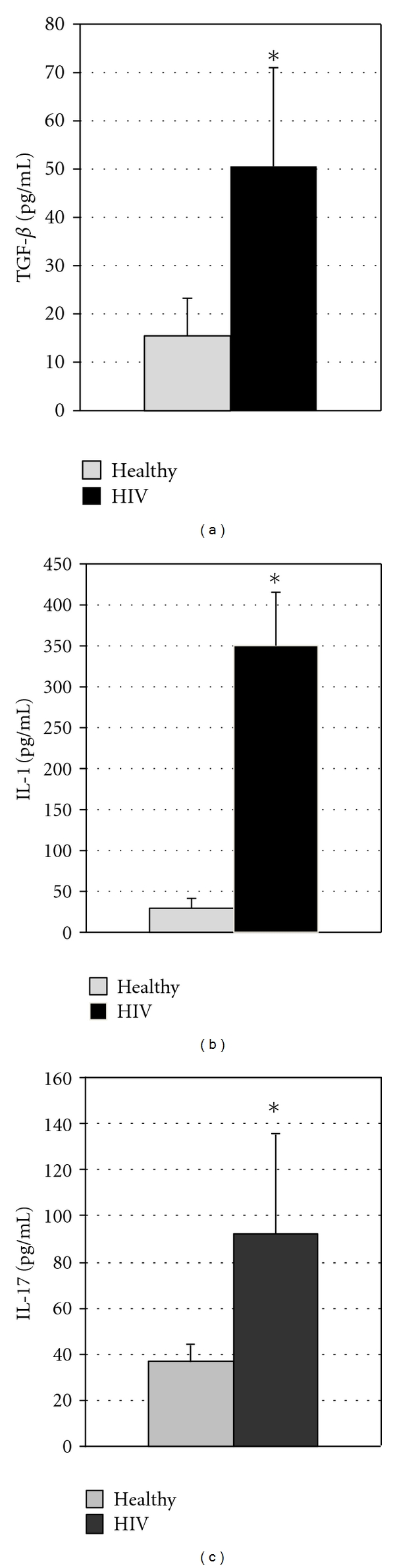
(a) *Assay of TGF-β in plasma from HIV-infected and healthy subjects*. Plasma samples separated from blood of healthy volunteers and HIV-infected individuals were used for measurement of TGF-*β*. Levels of TGF-*β* in the plasma samples were determined by ELISA using assay kits procured from eBioscience. Results in Figure 2 are averages of data collected from eight healthy subjects and twelve individuals with HIV infection. Results show elevated TGF-*β* in HIV-infected subjects (*P ≤ 0.05). (b) *Assay of IL-1 in plasma from HIV-infected and healthy subjects*. Plasma samples separated from blood of healthy volunteers and HIV-infected individuals were used for measurement of IL-1. Levels of IL-1 in the plasma samples were determined by ELISA using assay kits procured from eBioscience. Results in (b) are averages of data collected from eight healthy subjects and twelve individuals with HIV infection. Results show elevated IL-1 in HIV-infected subjects (*P ≤ 0.05). (c) *Assay of IL-17 in plasma from HIV-infected and healthy subjects.* Plasma samples separated from blood of healthy volunteers and HIV-infected individuals were used for measurement of IL-17. Levels of IL-17 in the plasma samples were determined by ELISA using assay kits procured from eBioscience. Results in (c) are averages of data collected from eight healthy subjects and eight individuals with HIV infection. Results show elevated IL-17 in HIV-infected subjects (*P ≤ 0.05).

**Figure 3:**
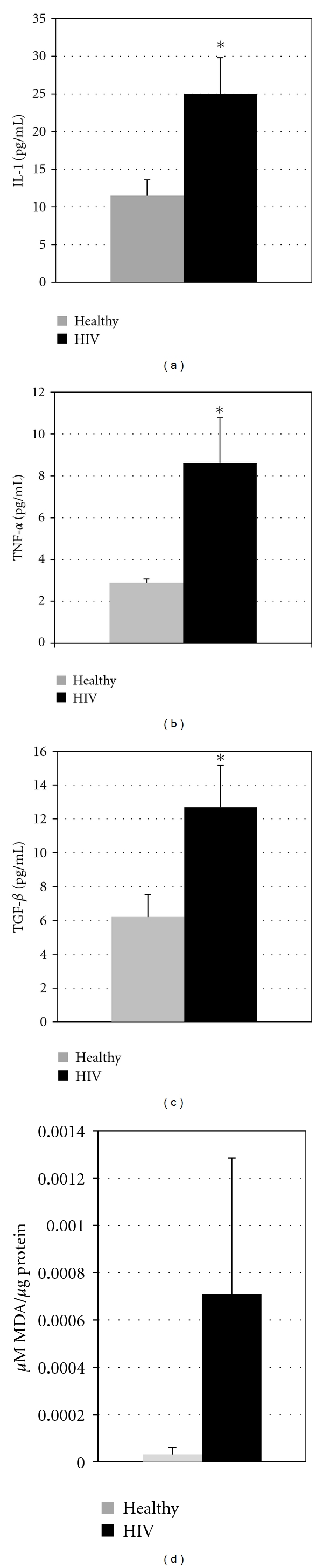
(a) *Assay of IL-1 in macrophage culture supernatants from HIV-infected and healthy subjects*. Supernatants from human monocyte-derived macrophages (from healthy and HIV-infected individuals) were assayed for the levels of IL-1 using assay kit from ebioscience. Data in (a) denote means ± SE from eight healthy individuals and nine individuals with HIV infection (*P ≤ 0.05). (b) *Assay of TNF-*α* in macrophage culture supernatants from HIV-infected and healthy subjects*. Supernatants from human monocyte-derived macrophages (from healthy and HIV-infected individuals) were assayed for the levels of TNF-*α* using assay kit from ebioscience. Data in (b) are means ± SE from eight healthy individuals and nine individuals with HIV infection (*P ≤ 0.05). (c) *Assay of TGF-β in macrophage culture supernatants from HIV-infected and healthy subjects*. Supernatants from human monocyte-derived macrophages (from healthy and HIV-infected individuals) were assayed for the levels of TGF-*β* using assay kit from ebioscience. Data in (c) represent means ± SE from eight healthy individuals and nine individuals with HIV infection (*P ≤ 0.05). (d) *Assay of MDA in macrophage lysates from HIV-infected and healthy subjects.* Free radical levels in macrophage lysates from healthy subjects and individuals with HIV infection were determined by measuring the levels of MDA using a colorimetric assay kit from Cayman. Results in (d) are averages of data collected from five healthy subjects and five individuals with HIV infection. These elevated MDA concentrations are indicative of elevated free radical concentrations. High standard error values observed in HIV-infected subjects (a–d) are likely due to the varying stages of HIV infection and antiretroviral treatment in the HIV-infected population.

Measurement of IL-1 concentrations in the plasma of healthy and HIV-infected individuals revealed a significant increase in the amounts of IL-1 present in the plasma of HIV-infected individuals over those found in healthy individuals. In fact, our study demonstrates a greater than 6-fold increase in the plasma IL-1 concentrations of HIV-infected individuals (Figure 2(b)). In addition, our assay of IL-1 in the culture supernatants of macrophages from HIV-infected and healthy individuals demonstrated a significant increase in the production of IL-1 by HIV-infected over uninfected macrophages (Figure 3(a)).

 We also observed a significant increase in the levels of TGF-*β* in the plasma of HIV-infected individuals compared to healthy subjects (Figure 2(a)). Assay of TGF-*β* in the supernatants of macrophage cultures also demonstrated a significant increase in the production of TGF-*β* by macrophages from the HIV-infected group over those from the healthy group (Figure 3(c)).

 Our analysis also included an assay of IL-17 in the plasma of our HIV and control groups. We found a significant elevation in the levels of IL-17 in the plasma from the HIV-infected group in comparison to the control group (Figure 2(c)).

 Our comparison of TNF-*α* in the supernatants of HIV-infected and healthy macrophages revealed a significant increase in TNF-*α* production by HIV-infected macrophages (Figure 3(b)).

 In addition to the cytokines mentioned above, we also assayed the concentrations of MDA in the macrophage lysates from healthy and HIV-infected subjects. MDA is a byproduct formed during lipid peroxidation. Measurement of MDA has been shown to be a reasonably accurate representation of free radical formation [13, 14]. Our assay of MDA revealed increased levels in the macrophage lysates of HIV-infected individuals over healthy individuals (Figure 3(d)). These elevated MDA levels correspond to elevated production of free radicals.

### 3.2. Assay of GSH in Macrophage Lysates

**Figure 4:**
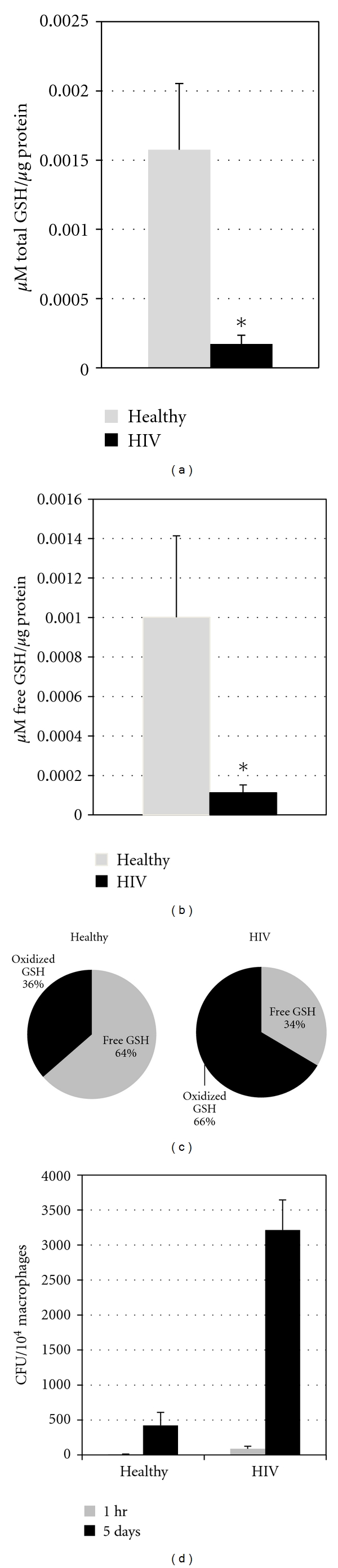
(a)* Assay of total GSH concentrations in macrophage lysates from HIV-infected and healthy subjects.* GSH levels were measured in isolated macrophages from healthy subjects and individuals with HIV infection by spectrophotometry using an assay kit from Arbor Assays. Briefly, an equal volume of ice cold 5% 5-SSA was added to the macrophage (3 × 10^5^) pellet. Supernatants collected after centrifugation were analyzed for total, and oxidized GSH as per manufacturer's instructions. All GSH measurements were normalized with total protein levels. Data in (a) represent means ± SE from five different healthy and HIV-infected individuals. GSH concentrations are decreased in HIV-infected macrophages with respect to healthy macrophages (*P ≤ 0.05). (b)* Assay of free GSH concentrations in macrophage lysates from HIV-infected and healthy subjects. *Free GSH was calculated by subtracting measured oxidized GSH concentrations from the measured total GSH concentrations, per the manufacturer's instructions. Data in (b) denote means ± SE from five different healthy and HIV-infected individuals. GSH concentrations are decreased in HIV-infected macrophages with respect to healthy macrophages (*P ≤ 0.05). Free GSH concentrations are decreased in HIV-infected macrophages with respect to healthy macrophages (*P ≤ 0.05). (c) A comparison of the composition of the total GSH in healthy and HIV-infected subjects reveals that the majority of GSH in HIV-infected macrophages exists as oxidized GSH (~70%), whereas healthy macrophages contain a more balanced GSH makeup (~40% oxidized, ~60% free). (d) *Intracellular growth of M. tb in macrophages from healthy and HIV-infected individuals at 1 hour, and 5 days postinfection*. Human monocyte-derived macrophages (from healthy and HIV-infected individuals) were infected with the processed H37Rv at a multiplicity of infection of 10 : 1. Infected macrophages were terminated at 1 hour and 5 days postinfection to determine the intracellular survival of H37Rv inside macrophages from healthy and HIV-infected individuals. Macrophage lysates were plated on 7H11 medium enriched with ADC to estimate the growth or killing of H37Rv. Results shown in (d) are averages from *n* = 4 HIV and *n* = 5 healthy. Each experiment was performed in triplicate. Macrophages from HIV-infected subjects demonstrate a markedly decreased ability to control intracellular *M. tb*  growth.

Our analysis of GSH in macrophage lysates demonstrated a marked decrease in the total GSH present in HIV-infected macrophages when compared to uninfected macrophages (Figure 4(a)). In addition, our analysis of the GSH present in the tested macrophage lysates demonstrated significantly low levels of free GSH in macrophages from individuals with HIV-infection when compared to macrophages from healthy subjects (Figure 4(b)). Of particular interest are the relative percentages of free GSH and GSSG. In macrophages isolated from HIV-positive subjects, we observed the total GSH as being composed of about 30% free GSH and 70% GSSG. In macrophages isolated from healthy individuals, we observed a GSH composition of about 60% free GSH and 40% GSSG (Figure 4(c)).

### 3.3. Determination of the Intracellular Survival of *M. tb* in Isolated Monocyte-Derived Macrophages

Our test for the intracellular survival of *M. tb* in isolated macrophages demonstrated growth of *M. tb* in the macrophages from both healthy and HIV-infected macrophages; however, there was several-fold increase in the growth of *M. tb* in macrophages from HIV-infected subjects. In fact, there was over 3 times the *M. tb* growth in HIV-infected macrophages when compared to healthy macrophages (Figure 4(d)). These results confirm that the ability of the macrophages collected from HIV-infected subjects to control infections is impaired.

### 3.4. Comparative Gene Expression Analysis of GSH Synthesis Enzymes

**Figure 5:**
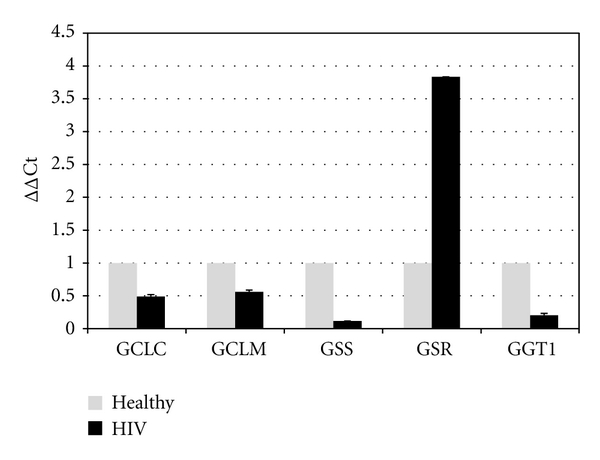
Relative gene expression for the specified target genes in comparison to the endogenous control ACTB. A comparison of gene expression in macrophages from healthy and HIV-infected subjects demonstrates reduced gene expression for all genes involved in the de novo synthesis of GSH in macrophages from HIV-infected subjects. A greater than 3-fold increase in GSR expression is observed in HIV-infected macrophages. Results are from *n* = 3 individuals for both healthy and HIV-infected macrophages.

Comparative gene expression analysis of GCLC, GCLM, GSS, and GGT1 in macrophages from HIV-infected subjects demonstrated a significant reduction in the expression of these genes when compared to healthy macrophages. Expression of GCLC and GCLM in HIV-infected macrophages was reduced by about half. Expression of GSS in HIV-infected macrophages was reduced by about 89%. Interestingly, GSR expression in HIV-infected macrophages was increased by a factor of 3.8 over GSR expression in macrophages isolated from healthy subjects. Finally expression of GGT1 in HIV-infected macrophages was found to be reduced by about 80% compared to healthy macrophages (Figure 5).

## 4. Discussion

*M. tb* remains one of the most pernicious and an enduring pathogen of mankind. It is inferred that the first infected cell is the alveolar macrophage that internalizes the bacilli following inhalation of droplets aerosolized by infected individuals [15]. The macrophage is known to be the primary defense mechanism against microbial invasion [16, 17]. Macrophages have a phagocytic system that delivers the microbe into a compartment, that is, the site of generation of reactive oxygen intermediates, increasing acidity, hydrolytic activity, and the presence of antimicrobial peptides [15]. Importantly, we reported that GSH plays a key role in limiting intracellular growth of H37Rv in both human and murine macrophages [4–6]. Thus, GSH has direct antimycobacterial activity distinct from its role as an NO carrier, functioning as an effector molecule in innate defense against *M. tb* infection [4–6]. These results unfold a novel and potentially important innate defense mechanism adopted by human macrophages to control *M. tb* infection [5, 6]. Consistent with these observations, we have also found that GSH in combination with cytokines, such as IL-2 and IL-12, enhance the activity of natural killer cells to control *M. tb* infection inside human macrophages [7]. Importantly, data from our most recent studies indicate that GSH activates the functions of T lymphocytes to control *M. tb* infection inside human monocytes (unpublished). All these observations support the fact that GSH controls *M. tb* infection by functioning as an antimycobacterial agent as well by enhancing the functions of immune cells. Finally, we demonstrated that GSH levels are significantly reduced in PBMC isolated from individuals with HIV infection, and this decrease correlated with increased production of proinflammatory cytokines and enhanced growth of *M. tb* [8].

 In this study, we examined the causes for decreased GSH in individuals with HIV infection. Furthermore, we also characterized the effects of decreased GSH in impairing the growth inhibition of *M. tb* inside macrophages.

 Measurement of IL-1 and TGF-*β* concentrations in the plasma and macrophage supernatants of healthy and HIV-infected individuals revealed a significant increase in the amounts of these cytokines present in HIV-infected individuals over healthy individuals. IL-1 is a proinflammatory cytokine, when bound to its receptor, and transduces a signal that initiates expression of a wide variety of inflammatory genes via the NF-*κ*B system. The subsequently transcribed genes can produce a variety of inflammatory products including chemokines, and proinflammatory cytokines, such as TNF-*α*, IL-6, or IL-8 [18]. IL-1 and TNF-*α* have been shown to be produced by either the binding of gp120 to the CD4 molecules on mononuclear phagocytes or infection with HIV [19, 20]. Several studies have demonstrated a link between increased TGF-*β* production and decreases in GCLC gene expression, as well as synthesis of GSH [21, 22]. In addition, we observed significant increases in the production IL-1 and TGF-*β* in the supernatants of macrophage cultures from the HIV-infected group compared to healthy individuals (Figures 3(a) and 3(c)).

 We also observed a significant increase in the levels of IL-17 in plasma samples from individuals with HIV-infection. IL-17 is thought to play a significant role in activating and inducing antimicrobial peptides and proinflammatory cytokines like IL-6, CCL2, and TNF-*α* [23, 24]. Furthermore, high levels of this cytokine have been linked to a number of inflammatory diseases including rheumatoid arthritis, multiple sclerosis, and asthma. Low levels, on the other hand, are thought to cause both impaired host defense against mycobacterial infection and decreased antibacterial immunity [23, 25].

 Studies on the effects of HIV on IL-17 concentrations using flow cytometry have found that HIV-infected patients have significantly increased levels of IL-17 [26]. However, Brenchley et al. [25] noted that there were significantly fewer IL-17 producing Th17 cells in the gastrointestinal tract of HIV-infected patients. In fact, the study indicated that Th17 cells were preferentially targeted during HIV infection. The decrease of IL-17 concentrations at the mucosal wall of the gastrointestinal tract could greatly increase the probability of bacterial infections, which could in turn have significant implications for the speed of HIV pathogenesis [26]. As Levy noted [27], chronic immune activation increases the production of proinflammatory cytokines (IL-6, IL-17, TNF-*α*, etc.). This upregulation of proinflammatory cytokines often leads to the rapid loss of CD4^+^ T cells via apoptosis.

 We observed a significant increase in the production of TNF-*α* in the supernatants of macrophage cultures from the HIV-infected group compared to healthy individuals (Figure 3(b)). Moreover, in a separate study, we have observed significantly increased concentrations of TNF-*α* in the plasma of HIV-infected subjects (unpublished). TNF-*α*, another proinflammatory cytokine which plays a major role in HIV infection, is produced by monocytes, macrophages, and natural killer cells in response to HIV infection [19, 20]. There is correlation between HIV viremia and TNF-*α* and IL-1 levels in the serum of HIV-infected patients. Excess production of TNF-*α* may lead to inflammatory damage and toxicity, which could lead to degradation of the host immune response independent of CD4^+^ T cell depletion. These high levels of TNF-*α* contribute to fever, anorexia, and other symptoms of HIV/AIDS. As HIV causes chronic infection, this leads to the prolonged production of IL-1 and TNF-*α*, which stimulate the prolonged production of free radicals. This chronic state of inflammation leads in turn to chronic oxidative stress [19, 20].

 Our assay of MDA revealed increased levels in the macrophage lysates of HIV-infected individuals over healthy individuals (Figure 3(d)). These elevated MDA levels correspond to elevated production of free radicals. Furthermore, in a previous study, we demonstrated significantly elevated levels of MDA in plasma isolated from HIV-infected individuals over plasma from healthy individuals (unpublished).

 We observed a significant decrease in both the total and free GSH present in macrophages from individuals with HIV infection compared to macrophages from healthy subjects (Figures 4(a) and 4(b)). We also observed an increased percentage of GSSG in macrophages from individuals with HIV infection compared to macrophages from healthy subjects (Figure 4(c)).

 The decrease in the levels of both total and free GSH correlated with several-fold increase in the growth of *M. tb* inside macrophages from individuals with HIV infection (Figure 4(d)). These results confirm that the ability of the macrophages derived from HIV-infected subjects to control *M. tb* infection is impaired. The immunocompromised status of the HIV-infected macrophages correlates with the increased levels of proinflammatory cytokines, TGF-*β*, and decreased levels of GSH.

 Chronic oxidative stress is commonly observed in HIV patients, indicating a benefit for increased antioxidant supplements which may reduce DNA damage, possibly slowing the progression of infection [28]. The progression of HIV infection is characterized by a loss of CD4^+^ T cells and decreased immunity. Prolonged free radical overload of monocytes and granulocytes in combination with the decreased production of GSH synthesis enzymes indicated by our experiments contribute to the deficiency of antioxidant mechanisms, GSH in particular. This may lead to the loss of CD4^+^ T cells often seen in the progression of HIV [28–30].

 We observed a significant reduction in the expression of GCLC, GCLM, GSS, and GGT1 genes in macrophages from individuals with HIV infection compared to healthy subjects. As most cells are unable to transport GSH or GSSG across the plasma membrane, these compounds must be first cleaved at the *γ*-glutamyl bond before they can be transferred into the cytosol [10, 31]. GGT1 performs this cleavage, allowing the components for new GSH molecules to be transferred into the cell [11, 12]. The reduction in GGT1 expression we observed would indicate a reduction in the raw materials necessary for de novo GSH production, also contributing to GSH deficiency. Interestingly, GSR expression in HIV-infected macrophages was increased by a factor of 3.8 over GSR expression in macrophages isolated from healthy subjects. This increased GSR expression indicates an attempt by the cell to increase free GSH concentrations through the reclamation of oxidized GSH. However; since the productivity of this enzyme is limited by the amount of oxidized GSH present and requires NADPH as a cofactor, this increase in GSR does not appear to be enough to compensate for the decrease in de novo GSH production.

To summarize, we observed a significant increase in the levels of proinflammatory cytokines such as IL-1, TNF-*α*, and IL-17 in individuals with HIV infection (Figures 2 and 3). This increase in the levels of proinflammatory cytokines in individuals with HIV infection correlated with increased production of free radicals (Figure 3). Additionally, we also observed a significant increase in the levels of TGF-*β* in both plasma and macrophage supernatants from individuals with HIV infection, and this increase correlated with reduction in the expression GCL gene in macrophages (Figures 2, 3, and 5). The results of our studies indicate that increased levels of free radicals (induced by proinflammatory cytokines) and decreased expression GCL (induced by TGF-*β*) may cause decrease in the levels of GSH in macrophages derived from individuals with HIV infection. Importantly, decreased levels of GSH in macrophages from individuals with HIV infection is accompanied by enhanced intracellular survival of *M. tb*.

**Figure 6:**
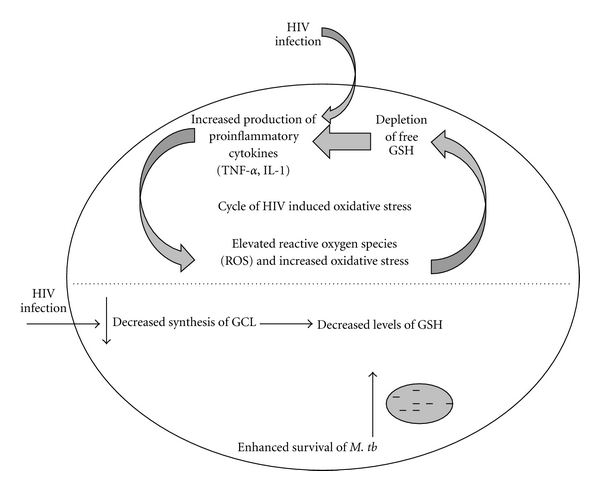
Model illustrating the reasons for decreased levels of GSH in macrophages from individuals with HIV infection.

 To conclude, we observed a correlation between decreased intracellular GSH levels, increased proinflammatory cytokines, and increased free radicals. Our data supports our hypothesis that decreased intracellular GSH levels in HIV-infected individuals is a result of chronic overproduction of inflammatory cytokines (IL-1, TNF-*α* and IL-17), and cytokines (TGF-*β*) which interfere with the biosynthesis of GSH (Figure 6). Our data also supports the idea that chronic inflammation leads to increased production of free-radicals, which in turn promote production of proinflammatory cytokines, and thus further depletion of intracellular GSH, and increased production of free radicals. We believe that this is a self-promoting loop of inflammation. In future studies, we hope to demonstrate that this loop can be broken by supplementing GSH. If successful, our data would indicate the possibility of efficacy for supplemental GSH therapy in individuals with HIV and *M. tb *coinfection.
